# Mathematical method for physics-based rill erosion process using detachment and transport capacities

**DOI:** 10.1038/s41598-022-08512-6

**Published:** 2022-03-21

**Authors:** Y. Y. Ban, T. W. Lei

**Affiliations:** 1 Key Laboratory of Water Cycle and Related Land Surface Processes, Institute of Geographic Sciences and Natural Resources Research, Chinese Academy of Sciences, Beijing, 100101 People’s Republic of China; 2 China Agricultural University, Beijing, 100083 People’s Republic of China

**Keywords:** Hydrology, Solid Earth sciences, Mathematics and computing

## Abstract

Quantification of rill erosion processes is of great importance in both model parameter estimations for process-based rill erosion models and in model performance verification. This study presents a mathematical method to determine a physics-based rill erosion process derived from the feedback relationship of transport capacity and detachment capacity. Experimental data sets were used to determine transport capacities under steep slope gradients of 15°, 20°, and 25° and the detachment capacities. The estimated transport and detachment capacities were then used to determine the sediment delivery processes under different hydraulic regimes. The sediment concentrations along the rill determined with the mathematical method were compared with the experimental measurements to verify the methodology and the mathematics. Results showed that the mathematical model results agreed well with the experimental data in references. The predicted detachment capacity calculated by the new method was capable of predicting saturated, unsaturated, and thawed slope, but incapable of partially thawed soil. This study not only supports the analytical solution to the differential equation of rill erosion, but also verifies that the experimental method was fit well with the mathematical concept. The new method provides a useful and efficient way to quantify rill erosion processes.

## Introduction

Rill erosion is closely related to serious soil erosion and therefore reinforcing soil degradation and threatening agricultural development^1^, which has received widespread concern from both farmers and researchers. Researchers develop soil erosion model to better predict sediment process and attempt to control land degradation. Soil erodibility and sediment transport capacity are important parameters to quantify soil erosion extent and the resistance to shear deformation by runoff^2^.

The soil erodibility indexes are defined by increase of detachment with unit shear stress. To calculate detachment rate, researchers have worked hard by experiment and mathematical derivation which includes analytical and numerical methods. The analytical computation calculates detachment rate by relationship between sediment concentration and rill length^3,4^, but there is systematic error due to high value discrepancies^5^. The empirical computation estimates detachment rate by experimental data^6^, and its systematic error can be reduced by modified numerical method^7^.

The soil erodibility parameters are crucial to research soil erosion process, that researchers have made much efforts to determine them. The soil erodibility equation proposed by Gilley et al. (1993) is determined by experimental data that provides reliable estimate. Some researchers believe that soil erodibility keeps unchanged^2^.

In the development of process-based erosion models, one needs to understand the relationships among soil detachment, sediment transport, and deposition^8^. New experimental and computational methods to quantify rill erosion processes are needed, which are of great importance for developing physically based hillslope soil erosion models. The rill erosion processes in the WEPP model (Water Erosion Prediction Program)^9,10^ are essential components.

The detachment capacity and transport capacity coupled effects of suspended sediment on rill detachment were proposed by Foster and Meyer (1972)^11^ as:

1 $$\frac{{D_{r} }}{{D_{c} }} + \frac{{T_{r} }}{{T_{c} }} = 1$$

where *D*_*r*_ and *D*_*c*_ are the rill detachment rate and detachment capacity, per unit area of the rill channel bed during a unit time (kg s^−1^ m^−2^), respectively; *T*_*r*_ is the rill sediment load (kg m^−1^ s^−1^); and *T*_*c*_ is the sediment transport capacity of the water flow (kg m^−1^ s^−1^) in the rill.

In Eq. (1), *D*_*c*_ and *T*_*c*_ are two constant parameters at a location and/or a point in time, which are determined by the properties of the soil before it is scoured to become sediments and by those of the sediments after being removed from the soil body. The former depends on the detachment capability of the water flow, and the latter depends on the sediment transport capacity^12^.

When rill sediment load *T*_*r*_ = 0, Eq. (1) produces *D*_*r*_ = *D*_*c*_, which means the detachment rate is at its highest value at the detachment capacity. This defines a concept with which soil detachment capacity is determined. When *T*_*r*_ = *T*_*c*_, Eq. (1) produces rill detachment rate *D*_*r*_ = *0*. This means that the sediment delivery rate is the highest, and reaches sediment transport capacity. This conceptually gives a method to determine sediment transport capacity. Between these two extreme conditions, the lowest sediment concentration and the highest possible sediment concentration at sediment transport capacity, higher sediment loads, *T*_*r*_, in the water flow either reduce soil detachment rates or induce sedimentation (deposition), *D*_*r*_.

Rearranging Eq. (1) produces the following equation:

2 $$D_{r} = D_{c} \left( {1 - \frac{{T_{r} }}{{T_{c} }}} \right)$$

Equation (1) clearly indicates that *D*_*c*_ and *T*_*c*_ are essential in describing the rill erosion process. Once these two key parameters are defined, the rill erosion process can possibly be determined or estimated. These parameters are known to be difficult to obtain and calculate, and the theoretical concept definitely also needs to be verified with suitable data sets produced from proper experimental configurations. Sediment transport capacity is the extreme value of sediment concentration of a given hydrodynamics condition. It can be experimentally determined with experiments when a rill is sufficiently long, and can produce steady hydrodynamics condition. This hypothesis for experiments under given conditions needs to be proven, with experimental results to support.

Scientists have made great efforts to determine sediment detachment capacity and transport capacity to better understand the rill erosion process. However, no widely acceptable measurement techniques for these capacities have been established yet^13^. Nearing et al. (1989) reported that detachment rate by sediment laden water flow along a rill is a function of detachment capacity to soil erodibility, shear stress, sediment load and sediment transport capacity of the water flow^14–16^. Then they related soil erosion rates to stream power and obtained the function parameters by regression of laboratory experimental data from rill flow studies^17^. This expression was proven to be applicable to sediment transport capacity^18^, and they improved a model to simulate the dynamic process of rill evolution and give a better understanding of the rill erosion process. This dynamical and spatial variation model simulated the evolution of the rill morphology as a dynamic function of the erosion process. However, due to limitations such as gentle experimental slopes, and rills with varying widths, more studies were needed.

In the present study, a mathematical model using regressed experimental data based on the WEPP erosion science has been developed. This model represents the relationships between sediment load processes and rill lengths at different slopes and flow rates. The rill erosion process perfectly fits with the measurements and modeled values. Validation of this type has not been conducted before and the mathematical model statistics could not explain the meaning of the parameters. Thus, the physical significance of the parameters remains unknown. As the importance of parameters in soil erosion model, the detachment capacity is meaningful for soil erosion process research. Although existed research predicted detachment rate and capacity with different methods, the error of empirical equation and the correctness remain to be proved.

In recent research, the determination of sediment transport capacity remains crucial and difficult, especially for gentle slopes. Normally, experimental facilities used to create rills are not long enough to generate sediment concentrations in the water flow that can reach its extremum values. By contrast, shorter rill lengths on non-erodible steeper slopes allow flows to more easily reach the sediment transport capacity. Therefore, usually only the rill erosion processes on steeper slopes can be used to verify rill erosion processes determined with transport capacity and detachment capacity to clarify the physical meanings of the rill erosion process function. An analytical solution to the differential equation of rill erosion was presented, that clearly presented the relationship between sediment concentration and rill length^4^. However, they did not verify the equation of the analytical solution.

This study aims to: (1) present a mathematical method to determine a physics-based rill erosion process derived from the feedback relationship of transport capacity and detachment capacity; (2) determine parameters needed in quantifying the rill erosion processes by experimental data; (3) determine the transport and detachment capacities through sediment delivery processes; and (4) verify the methodology and the mathematics by experimental data in references.

## Mathematical method for physics-based rill erosion process

### Traditional estimation method for detachment rate

The rill erosion component in the WEPP model uses a steady-state sediment continuity equation to quantify the soil detachment rate in a rill, and includes detachment and transport coupling. The soil detachment rate^14^ is given by the following equation:

3 $$D_{r} = K_{r} \left( {\tau - \tau_{c} } \right)\left( {1 - \frac{qc}{{T_{c} }}} \right)$$

where *K*_*r*_ is the rill erodibility parameter (s/m), *τ* is the shear stress of the flowing water (Pa), *τ*_*c*_ is the critical shear stress of the soil (Pa), *q* is the unit-width flow rate (m^3^ m^−1^ s^−1^), and *c* is the sediment concentration in water flow (kg m^−3^). The product *qc* is also known as the sediment load of the flow. Equation (3) is applicable when sediment load is less than sediment transport capacity (i.e., *qc* < *T*_*c*_).

Deposition is predicted to occur when the sediment load in the water flow is greater than the transport capacity (i.e. *qc* > *T*_*c*_), then *D*_*r*_ is negative and a different equation for rill deposition is used^14^. The sediment transport capacity, critical shear stress, and soil erodibility all depend on the interactions between the soil and the water flow. They are essential parameters for rill erosion prediction.

When the shear stress of flowing water is greater than the critical shear stress of the soil to withstand it, the water flow reaches an energy level to detach soil particles from the rill bed. When clear water (i.e., *c* = 0) is introduced into a rill, the detachment rate will be at its maximum, or at the “detachment capacity”, which is expressed as follows^14^:

4 $$D_{c} = K_{r} \left( {\tau - \tau_{c} } \right)$$

Substituting Eq. (4) into Eq. (3) yields

5 $$D_{r} = D_{c} \left( {1 - \frac{qc}{{T_{c} }}} \right)$$

The basic assumption for the estimation of detachment rate along a rill is that the soil detachment behavior and the hydrodynamic conditions are uniformly distributed along the rill under steady flow. This assumption means shear stress down a rill will typically increase with distance, thus $D_{c}$ will increase spatially with distance downslope before reaching sediment transport capacity. The requirement for non-change of $T_{c}$ spatially and temporally needs steady water flow to be used in the experiments. Under this assumption, if sediment load produced from a rill of $x$ is $c$, and that from a rill of $x + dx$ is $c + dc$, then the increment in sediment concentration, $dc$, represents the sediment particles detached from the rill segment length of $dx$. This study only formulated these equations for rill flow and detachment, with no interrill contributions. This can be quantitatively computed as follows:

6 $$D_{r} = q\frac{dc}{{dx}}$$

where *x* (m) is the position downslope along the rill length.

Substituting Eq. (5) into Eq. (6) produces the following:

7 $$q\frac{dc}{{dx}} = D_{c} \left( {1 - \frac{qc}{{T_{c} }}} \right)$$

### Empirical and modified empirical method for detachment rate

The transport capacity is directly related to the maximum sediment concentration that water flow can transport, as given by the following:

8 $$T_{c} = qc_{\max }$$

Substituting Eq. (8) into Eq. (7) yields the following:

9 $$\frac{dc}{{c_{\max } - c}} = \frac{{D_{c} dx}}{{T_{c} }}$$

The solution of the differential equation, Eq. (9), requires that one boundary condition is specified, which is given as *c* = 0 when *x* = 0. This mathematical boundary condition specifies the physical meaning that clean water should be applied at the initial point of the rill. Integration of Eq. (9) is given as follows:

10 $$\int_{0}^{c} {\frac{dc}{{c_{\max } - c}}} = \int_{0}^{x} {\frac{{D_{c} }}{{T_{c} }}} dx$$

Solving Eq. (10) yields the following:

11 $$\ln \left( {c_{\max } } \right) - \ln \left( {c_{\max } - c} \right) = \frac{{D_{c} }}{{T_{c} }}x$$

Then, the relationship between sediment concentration and rill length is given as:

12 $$c = c_{\max } \left( {1 - e^{{ - \frac{{D_{c} }}{{T_{c} }}x}} } \right)$$

or

13 $$qc = T_{c} \left( {1 - e^{{ - \frac{{D_{c} }}{{T_{c} }}x}} } \right)$$

Equation (12) or (13) is a mathematical solution to the differential equation for rill detachment rate. But this equation quantifies the distribution of sediment concentration along a rill, which is directly related to or coupled with the rill detachment rate, as defined by Eqs. (6) or (7). This equation, Eqs. (12) or (13), clearly presents the physical meaning of the rill erosion process, related to sediment distribution along a rill as the result of rill detachment. When *D*_*c*_ in Eq. (13) is greater in value due to a greater rill erodibility of the soil, this would cause a faster increase in the sediment concentration in the flow along the rill distance. For a given soil with a certain detachment capacity, a greater *T*_*c*_ value results in a greater sediment deficit as related to *c*_*max*_ or *T*_*c*_, which means more sediments are needed before the rill flow reaches its maximum sediment concentration. Increase in the sediment concentration is less rapid under a condition of lower sediment transport capacity. Therefore, the mathematical expression of rill erosion in Eq. (13) clearly presents the physical meanings of the parameters in the rill erosion process.

According to the hydrodynamic conditions of rill erosion and the energy inversion theory, a series of laboratory experiments were designed to study the dynamic rill erosion processes^4^. An empirical mathematical model was derived from that study to quantitatively represent the experimental data obtained as follows:

14 $$c = A\left( {1 - e^{ - Bx} } \right)$$

where *A* is a regression coefficient, representing the maximum potential sediment concentration (kg m^−3^), and *B* is an exponential decay coefficient (m^−1^).

The relationship between sediment concentration and rill length for the slope gradients and the inflow rates measured in the study^4^ were analyzed in the current study using the process-based equations presented above. Equation (13), the process-based equation, and Eq. (14), which is empirically derived from the experiments, both represent the same physical phenomenon and should agree with each other. The parameters in Eq. (13) bear physical meanings; however, Eq. (14) is only an empirical equation that fits the experimentally measured sediment distribution along an eroding rill. The identity of the two equations needs the verification of the following:

15 $$\left\{ \begin{gathered} A = c_{\max } \hfill \\ B = \frac{{D_{c} }}{{T_{c} }} \hfill \\ \end{gathered} \right.$$

Equation (15) indicates that the highest possible sediment concentrations from the two methods should be equal. The *c*_*max*_ in Eq. (12) is the measured maximum sediment concentration through the experiments, and *A* in Eq. (14) is the regressed maximum sediment concentration representing the highest possible sediment concentrations. The *B* value represents that the decreasing rate of sediment concentration increases along the rill. The term *D*_*c*_*/T*_*c*_ in Eqs. (12) and (13) provides physical meaning of the parameters and influencing factors. This term serves the same function as that of the empirical parameter *B* in Eq. (14). When *T*_*c*_ is greater (thereby resulting in a lower ratio of *D*_*c*_*/T*_*c*_ for a given *D*_*c*_ value), a longer rill is needed for the sediment concentration to reach the maximum value. When *D*_*c*_ is higher or the ratio of *D*_*c*_ to *T*_*c*_ is greater, a shorter rill will suffice for the sediment concentration to reach its maximum.

The physically based relationship between sediment concentration and rill length is represented as Eq. (13). The empirical model given by Eq. (14) was derived^4^ to fit the measured experimental data. Due to the limitations of the experimental method to reach transport capacity values under lower slopes of 5° and 10°, only the data sets at higher slope gradients of 15°, 20°, 25° were available. The parameters *c*_*max*_, *D*_*c*_*/T*_*c*_, *A*, and *B* are presented in Table 1.

**Table 1:** Parameters of Eqs. (10) and (11) under experimental slopes and flow rates from Lei et al. (2002).

Slope (°)	15°			20°			25°		
Flow rate (L/min):	2	4	8	2	4	8	2	4	8
A	780	760	760	850	800	850	820	850	870
*c*_*max*_ (kg/m3)	770	760	810	860	830	880	850	880	870
B	0.40	0.37	0.45	0.52	0.51	0.51	0.50	0.51	0.55
*D*_*c*_/*T*_*c*_ (1/m)	0.35	0.37	0.34	0.44	0.46	0.42	0.49	0.51	0.47

The values of *c*_*max*_ were directly obtained from the experimental data set as the maximum values of the measured sediment concentration, and *T*_*c*_ was computed as *qc*_*max*_. The transport capacity *T*_*c*_ was directly proportional to the maximum sediment concentration *c*_*max*_. The detachment capacity *D*_*c*_ was either numerically computed by Eq. (16) as follows, or analytically by Eq. (17):

16 $$D_{c} \approx D{}_{c}^{^{\prime}} = q\frac{\Delta c}{{\Delta x}}$$

In the above Eq. (16), sediment concentration $c$ in the water flow is at the rill position of *x*. When the rill length increases by *∆x*, the increase in sediment concentration is *∆c*. The detachment rate *D*_*c*_ can be estimated by Eq. (16).

The maximum detachment rate is acquired from Eq. (7) when the sediment concentration in the water flow is zero or at the initial position when *x* = 0. This is analytically computed through differentiating Eq. (14):

17 $$D_{c} = \left. {q\frac{dc}{{dx}}} \right|_{x = 0} = qAB$$

Substituting Eq. (14) into Eq. (16) yields the parameters of *D*_*c*_^*’*^ and *D*_*c*_:

18 $$D_{c}^{^{\prime}} = D_{c} \frac{1}{\Delta xB}e^{ - Bx}$$

19 $$D_{c} = D_{c}^{^{\prime}} \Delta xBe^{Bx}$$

**Figure 1:**
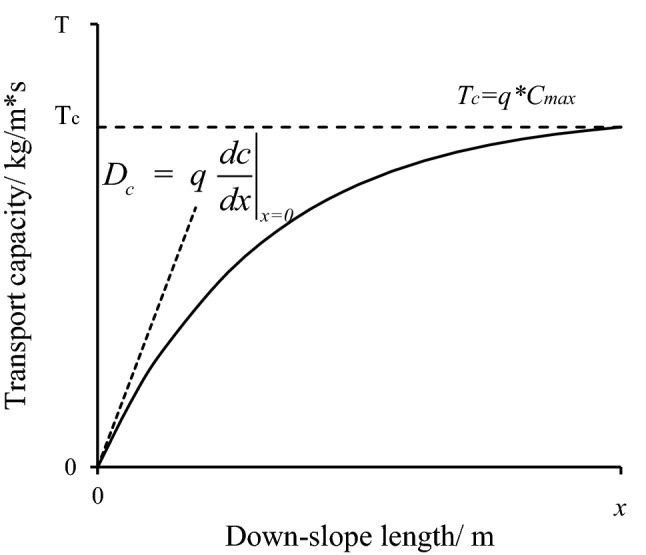
Calculation principle of transport capacity (*T*_*c*_) and detachment capacity (*D*_*c*_).

The numerical method, as described in Eq. (16), was referred to as the empirical method (EM). The method defined by Eq. (17) was the analytic method (AM). Rationally, the parameter value of *D*_*c*_ in the analytic method is the gradient of the tangent line of the sediment-distance function at *x* = 0, when the slope length is 0. And *T*_*c*_ is the sediment transport capacity determined by the maximum sediment concentration, as the limiting value when the rill length increases to a sufficiently long distance, as shown in Fig. 1. Equation (19) is the modified empirical method (MEM).

## Results and discussion

### Regression results between physically based equations and the empirical data

A silt loam (loess) soil was used in the experiment^4^, typical of the Chinese Northern Loess Plateau, with 20.2% sand (> 0.05 mm diameter), 63.9% silt (0.05–0.005 mm diameter), and 15.9% clay (< 0.005 mm diameter). The soil was packed into a flume 8 m long, 0.1 m wide, and 0.3 m deep, to a depth of 0.2 m and a bulk density of approximately 1.2 g cm^−3^. The soil was saturated 24 h before each experimental run to provide an evenly distributed initial water content and to eliminate the effects of uneven packing as much as possible. Sediment concentrations measured in the experiment were made at nine flow lengths of 0.5, 1.0, 2.0, 3.0, 4.0, 5.0, 6.0, 7.0 and 8.0 m, using three slope gradients and three flow rates. The slope gradients were 15°, 20°, and 25°, whereas the flow rates were 2, 4, and 8 L/min (i.e., 0.12, 0.24, and 0.48 m^3^ h^−1^). The detailed experimental method and procedures had been presented^4^.

To further check the validity of Eq. (17), the rill erosion processes estimated by the AM method were compared with those estimated by the MEM method. Linear regression analysis provided the proportionality coefficients and the coefficients of determination of the AM and the MEM methods, as listed in Table 2.

**Table 2:** Regression parameters between experimental data and mathematical model.

Slope (°)	Flow rate (L/min)	R^2^	Line Equation
15	1	0.939	Y = 1.02 x
	2	0.826	Y = 0.84 x
	4	0.907	Y = 0.95 x
20	1	0.848	Y = 0.86 x
	2	0.905	Y = 0.96 x
	4	0.918	Y = 0.97 x
25	1	0.892	Y = 1.07 x
	2	0.908	Y = 1.05 x
	4	0.909	Y = 0.95 x

Based on the comparisons of the results estimated by Eqs. (17) and (19) shown in Table 2, it is reasonable to conclude that the rill erosion process as defined by the sediment concentrations and estimated by the AM method agreed well with those by the MEM method. The relatively high coefficients of determination (R^2^) indicated good functionality of the AM method, as defined by Eq. (17). In addition, the AM model derived from the feed-back detachment approach in WEPP, represented by Eq. (17), was fully supported by the experimental data, indicating the feasibility of the experimental method. This condition indicated that for a given *T*_*c*_ value, greater detachment capacity of the flow/soil combination or higher soil rill erodibility produces more rill erosion^2^, which results in faster increases in the sediment concentration in the rill water flow. Under these conditions, sediment concentration in the water flow can reach its maximum with shorter length of rill^19^. The slope length needed for the flow to become saturated with sediments increases with the transport capacity of the water flow^20^. These results indicate that the AM and the MEM are available methods to predict *D*_*c*_ in rill erosion.

### Sediment load process

**Figure 2:**
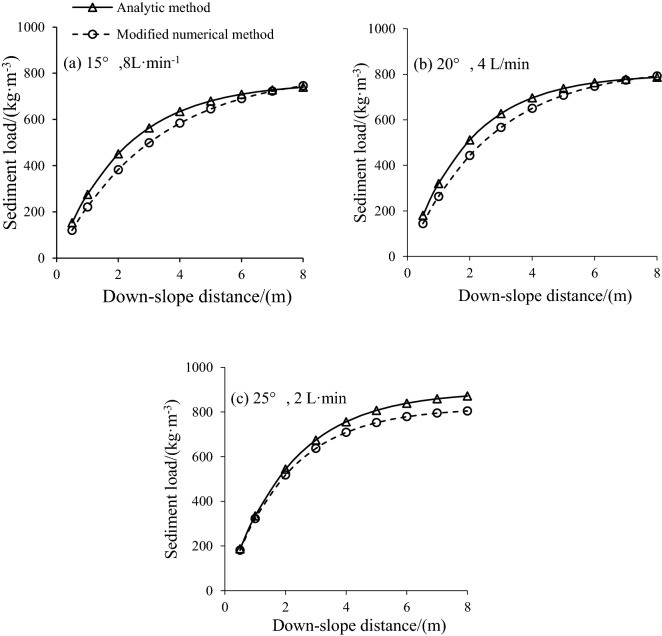
Comparisons of sediment distribution relationships by analytic method and the modified numerical method.

**Figure 3:**
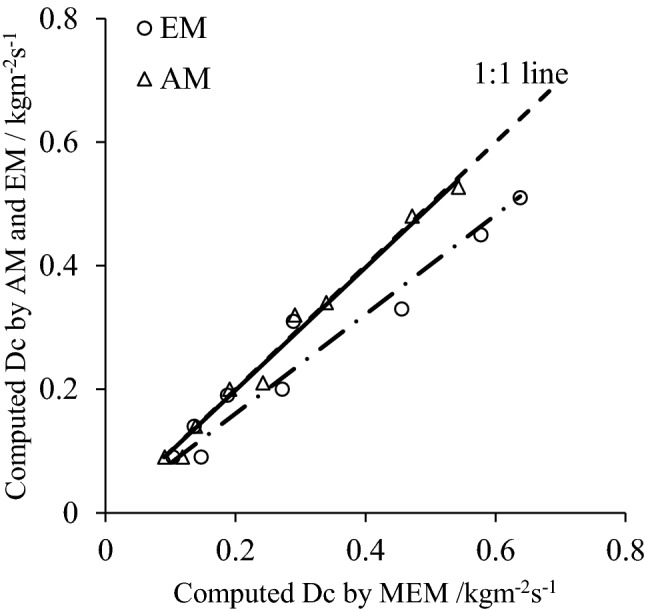
Computed result and comparison of *D*_*c*_ by different methods. EM is empirical method in Eq. (16), AM is analytic method in Eq. (17), and MEM is modified empirical method in Eq. (19).

The sediment-rill length relationships determined by the AM, Eq. (17), EM Eq. (16), and the MEM method, Eq. (19), under different slope gradients and flow rates are illustrated in Fig. 2. The results presented in Fig. 2a–c showed that these methods produced similar sediment load profiles. The sediment load initially rapidly increased with rill length, but the increase rate gradually decreased with downslope distance. For steeper slopes, the flume length required for the sediment concentration to reach its maximum was shorter compared to the lower slope gradients. The data shown in Fig. 2 indicated that the sediment loads, as a function of downslope distance estimated by the MEM method, were slightly greater than those from the AM method. These findings indicate the rationality and validity of the physical processes described by the analytic method. Rill detachment rate (Dr) gradually declined with rill length, but increased with sediment concentration^12,20^. The highest Dr appears if the water flow carries no sediment^21^. In Fig. 3, the Dc estimated by AM and MEM is much higher than AM of approximately 24%. The sediment nearly reaches its peak value from the mid-length and increase rate becomes much less henceforth^22^, which leads to the lower calculated Dc by EM.

The values of *D*_*c*_*/T*_*c*_ were estimated from the *D*_*c*_ and *T*_*c*_ data. *D*_*c*_ was computed by Eqs. (16), (17) and (19). The comparisons of the *D*_*c*_ values are presented in Fig. 3. The results shown in Fig. 3 indicate that the *D*_*c*_ values estimated with the MEM method (Eq. (19)) and the AM method (Eq. (17)) were almost identical. This verifies the correctness and validity of Eqs. (17) and (19). However, there were large differences between the *D*_*c*_ values computed with Eq. (16) and those with Eqs. (17) or (19). The proportionality coefficient of 0.803 illustrates that the *D*_*c*_ values calculated by Eq. (16) were substantially lower than those with Eqs. (17) or (19). The *D*_*c*_ and *T*_*c*_ play important role in modeling process based on soil erosion and researchers do their best to estimate these parameters^2^. However, these parameters are difficult to determine due to such as hydrological conditions, systematic errors, and experimental restrictions. Therefore, the MEM proposed in this research is meaningful for process-based soil erosion, and its application condition is explored as following.

### Model verification

**Figure 4:**
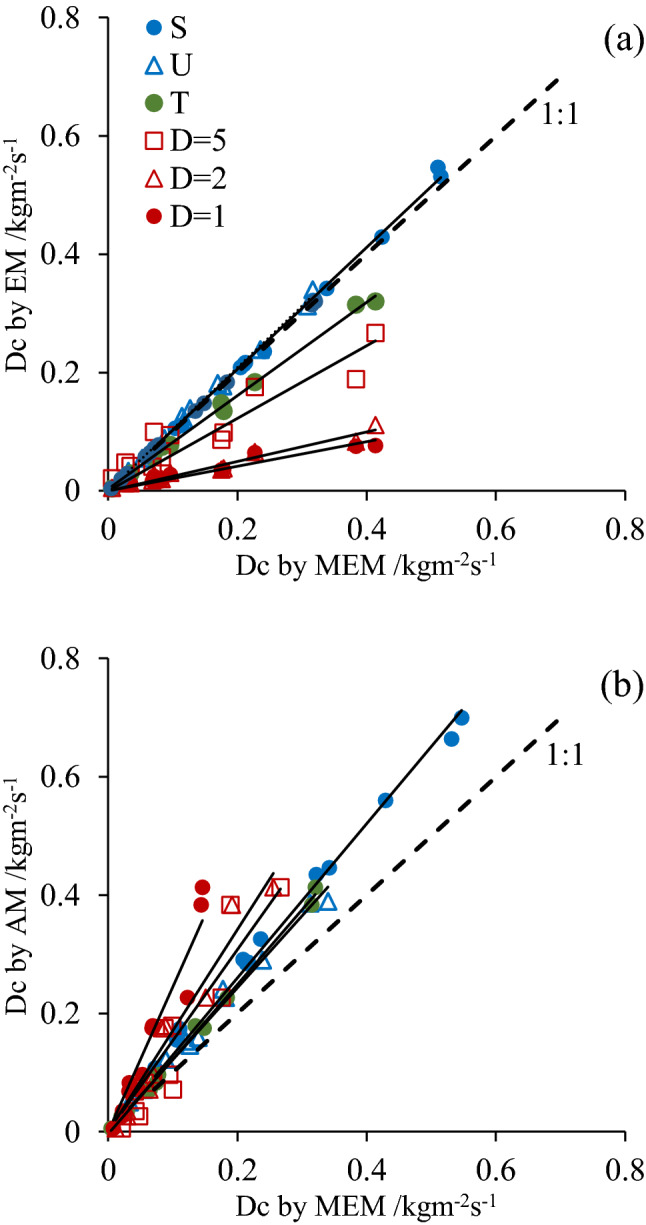
Comparison of Dc computed by AM, EM, and MEM from the experimental data in references. The legend in the figure is: S and U are saturated and unsaturated soil in^23^, T is thawed soil in^23^, D is thawed depth of frozen soil which is 1, 2, and 5 cm in^13^.

The regression model was verified by data from other research^13,20,23^. Their experimental data was introduced to verify the Dc calculation method as shown in Fig. 4. The overall results illustrated that Dc computed by EM and MEM are generally lower than AM, especially the frozen soil slopes.

Dc calculated in the reference^13,20,23^, showed the good linear correlation between EM and AM, MEM and AM, which indicated AM had high degree of accuracy to estimate Dc. The Dc in groups S and U in Fig. 4a was predicted by EM and AM indicating that soil water content had little influence on Dc computation, which is consistent with previous research^21^. However, the ratio between different groups in Fig. 4a suggested that EM was poor to predict Dc of shallower thaw soil on frozen slope. Insufficient sediment supplied by thawed soil with shallower depth increased slowly in water flow, however, less resistance and more energy made it had capacity to erode more sediment than the non-frozen or totally thawed soil^24^. The frozen status affected the sediment transport capacity of water flow, and in turn had direct impact on *Dc* computation^13^. Therefore, the EM over-underestimates Dc and is incapable of predicting Dc of thawed soil.

The EM, AM, MEM overestimated *Dc* by approximately 20% of saturated, unsaturated, and thawed soil, but by more than 70% of partially thawed soil, especially D = 1. It was verified that the modified numerical method had ability to compute detachment rate in rill of frozen slope^5^. In addition, the modified numerical method was useful for rill detachment rate of loess with different moisture content^7^. For partially frozen soil slope, thawed depth is a key factor significantly affecting soil erosion process^3^. Frozen layer controls rill morphology with less presence of headcuts^25^, and increases the impact of thawed depth on Dc estimation. Therefore, for the frozen surface, the AM can’t be applied to compute Dc, either.

## Conclusions

A mathematical method was deduced from the physically-based rill erosion concept to model the sediment concentration process along an eroding rill. The experimental data obtained from well-controlled flume experiments^4^ on steep slopes were used to illustrate the mathematical identity between the AM and the MEM. The sediment load processes along the eroding rill calculated by AM were compared with those calculated by MEM and produced similar results, showing that the regressed parameters bear clear physical meanings and the rationality of the MEM. The EM, AM, MEM overestimated Dc by approximately 20% of saturated, unsaturated, and thawed soil, but by more than 70% of partially thawed soil, which indicated that MEM was unavailable for frozen soil. This study provides a quantitative model of dynamic sediment transport processes. This result not only supported the validity of the MEM, but also demonstrated the feasibility of this method. Validation of three methods has been conducted and the mathematical model statistics could explain the meaning of the parameters. Thus, the physical significance of the parameters become clear. Additionally, further research is needed to verify MEM on different soil types and how to quantify Dc on gentle slopes especially with short length.
